# American Academy of Dental Science

**Published:** 1889-04

**Authors:** Thomas Fillebrown

**Affiliations:** Boston


### Reports of Society Meetings.

### AMERICAN ACADEMY OF DENTAL SCIENCE.

Report of Monthly Meeting, Wednesday Evening, February 6th, 1889.

### A NEW METHOD OF EXCISION OF THE INFERIOR DENTAL NERVE, WITH CASES.

BY THOMAS FILLEBROWN, M.D., D.M.D., BOSTON.

In connection with the operation which I wish to describe, I report two cases which have lately occurred in my practice, and in which this method of operating has proved a complete success in my hands.

Case I. Mr. H——, aged 56, in February, 1886, had his left inferior third molar extracted. (The first and second molars had been removed some years before.) At the time the operator thought that a fragment of the root was left in the jaw. Soon after this the patient became subject to attacks of pain in the left mental region, recurring at intervals. These spasms gradually increased in frequency and severity, and in October, when I first saw the patient, pain was rarely absent for more than five minutes at a time. The slightest irritation would cause the most intense pain. A current of air, warm or cold, contact of food with the teeth of the left side, or even the pressure of the lip by muscular contraction, would bring on these violent attacks. This long-continued suffering and inability to take proper nourishment showed their effects in the haggard, distressed look of his face and in his general physical condition. Aside from this the patient was apparently healthy.

Examination of the depression left by the extraction of the tooth revealed no evidence that a piece of the root had remained in the jaw; and the absence of tenderness, swelling, or redness indicated a normal condition. In one of the bicuspid teeth the pulp was dead, and treatment was directed to this in view of the possibility that the cause might lie here. Measures in this direction, however, had no influence over the pain. The failure of all treatment to afford relief to the sufferer satisfied me of the correctness of my first suspicions, namely, that the case was one of chronic inflammation of the inferior dental nerve. Excision remained the only proper course to pursue, which was readily agreed to by the patient.

Case II. Miss S——, aged 35, had been suffering several months from spasmodic neuralgia in left side of lower jaw and face, beginning with periodic attacks of slight pain, which gradually became so severe and frequent as to incapacitate her for work. Patient was anæmic, poorly nourished, and the nervous system showed a marked ıeaction to the long suffering. With the exception of the second and third molars the lower teeth of the left side were present and in good condition, offering no suggestion as to the cause of the pain. A general tonic treatment produced no appreciable effect.

Diagnosis: Chronic inflammation of inferior dental nerve, or pressure of tumor. Excision was decided upon.

In choosing a method of operating for these cases, it appeared to me that the nerve could be reached through the mouth, avoiding the ugly scar which results from the usual manner of operating from without through an incision along the ramus and body of the jaw. In the case of a lady especially this point is of much importance.

My plan was to cut down into the jaw through the site of the extracted teeth, and in this way expose the nerve and remove a section of the nerve through the opening. Confident of the practicability of such an operation, I determined to give it a trial, and as these cases will show the results were more than satisfactory.

The details of the operation were the same in both cases, therefore I have reserved their description for the sake of convenience.

The most important instrument used in the operation was made expressly for the purprse by Messrs. Codman and Shurtleff of Boston. The simplicity of the instrument is one of its principal features. It corresponds practically to the ordinary trephine, with the exception of the absence of the axis pin for fixation and its being adjusted to the dental engine.

In case I, the inferior molars of the left side being absent no teeth had to be extracted. Ether was given in the operating chair. The position of the head for operating was that which it is usually made to assume in ordinary dental operations upon the inferior teeth, namely: The head thrown somewhat forward and the chin approximated to the chest. Being so well accustomed to working under these circumstances, I found it unnecessary to use the gag, which really would have been more of a hinderance than a help. I removed a sufficient area of soft tissue immediately posterior to the second bicuspid. And having adjusted the trephine to the engine, which was managed by an assistant, the jaw was firmly supported from below with the left hand and the trephine was sunk perpen dicularly through the cortical and cancellous tissue until the plane of the nerve canal was supposed to have been reached. Directly behind, and in a line with this first opening a second and a third were made of the same depth.

The small pieces of the bone separated by the trephine were removed and the thin walls between the trephine holes broken with a large burr. Thus a longitudinal wound was made extending in the axis of the jaw, three-eighths of an inch wide and one inch in length.

Upon clearing away the blood clots and debris from the bottom of the opening, it was found that the nerve canal had not yet been reached. The trephine was again applied at each extremity of the wound, and sunk until the soft fleshy sensation transmitted to the hand signified that the nerve had been struck.

After removing the intervening bony tissue by means of the burr, a section of the nerve was then easily extracted, leaving the smooth, glistening nerve canal visible at the bottom of the cavity. After stopping the hemorrhage, which was slight, the wound was irrigated with a weak solution of carbolic acid. The operation was lengthened by the necessarily interrupted anæsthesia, and more in Case I, on account of the patient being addicted to the use of stimulants. The actual time of operation was about twenty minutes in each case. In Case I, the absence of three molars furnished abundant room in which to operate; but in Case II the presence of the first molar limited the field considerably. As regards the dressing of the wound, there is one point which I consider worthy of particular mention. The great difficulty with which wounds connected with the oral cavity are kept aseptic and dry after operation renders the treatment very unsatisfactory, and gives disappointing results.

The failure of my first dressing, iodoform and ordinary cotton tents, necessitated a substitute which would exclude the moisture, food and secretions of the mouth more effectually. Accordingly a pledget of cotton was taken, corresponding in size when slightly compressed, to the cavity in the jaw, and saturated with an alcoholic solution of gum sandarac. After having thoroughly dried the wound, and applying iodoform, this tampon was packed in forming a plug which filled up the cavity completely. The sandarac, of course, hardened immediately, and the wound was thus well protected from the food and fluids of the mouth. I found that this dressing worked admirably, and only required changing at intervals of two days in the beginning of the treatment, and later even after four days the wound would be found clean and free from disagreeable odor. At each dressing the tampon was made a little smaller. This dressing I would strongly recommend in the treatment of wounds of the mouth where its application is practicable. It forms a complete protection to the granulatory surfaces, and its application is very easy.

The results in these two cases were all that could be desired. Immediately after the operation in both instances the relief from pain was complete and permanent. There followed, naturally, insensibility and numbness of the teeth and face on that side; but that improved gradually in the course of time.

The excised portions of the nerves were examined by Dr. Irving Kimball, of Portland, and in the first case no pathological change was discovered, leaving the cause of trouble still very obscure. In the second case there was found a distinct tumor in the excised portion, with evidence of chronic inflammation in the surrounding nerve tissue.

In the operation which I have just described, one cannot fail to see the advantage over the external incision and chiseling the way to the nerve canal; and, without comparison, its merits are obvious to those who are accustomed to operate in the oral cavity. The only possible disadvantage in this method is the interrupted narcosis which, of course, lengthens the operation; but to my mind the avoidance of the disfigurement of the face and the salvation of the facial artery far outweighs this slight objection.

*President Wilson*—Gentlemen, you have no doubt all listened with a great deal of interest to Dr. Fillebrown's paper, and the subject is now open for discussion,

*Dr. Chandler*—How do you differentiate nerve ache from all the other aches that teeth are subject to?

*Dr. Fillebrown*—In the early stages of the trouble it is quite impossible to differentiate between them, but as the disease progresses the spasms of pain caused by the neuritis are more lancinating, more frequent, more intense, and also more completely intermittent than toothache.

Pain caused by a tooth is excited by only such extraneous circumstances as affect the teeth themselves while the neuralgia is excited by causes which affect only the surface as a current of air upon the face, or a slight touch as of a handkerchief even without pressure.

Tooth pain will generally sooner or later become located in some particular tooth which is the cause of it. Neuralgia persists in the mental region without any more definite localization.

Toothache seldom or never exhibits itself in the mental region. Neuralgias, very generally, if not always do.

Exclusion of the teeth as a cause by careful examination is an important matter in diagnosis.

*Dr. Wilson*—Are you aware of this operation ever having been performed inside the mouth before, or is it your own idea?

*Dr. Fillebrown*—It has been excised at its entrance into the posterior dental foramen, but never to my knowledge through the site of the molar teeth.

*Dr. Chandler*—How long a piece of nerve did you take out?

*Dr. Fillebrown*—About one inch.

*Dr. Wilson*—After having excised the nerve, what was the effect upon the other teeth in the lower jaw?

*Dr. Fillebrown*—The teeth were not changed in any respect observable.

*Dr. Brackett*—It seems to me that the paper which has been read before us describes an operation which is fairly to be considered a new operation, and that very high credit should be given to Prof. Fillebrown. Of course, we have all well known for years of the surgeon's procedure of removing a section of the inferior dental nerve by exterior operation; but to me the external operation seems comparable to this, as the external splint for fractured inferior maxilla is comparable to the Gunning interdental splint, or as lithotomy is camparable to litholopaxy. Vesical calculi had been removed through external incision from very early days in the history of surgery. Indeed, stones had been broken up in the bladder, and by a succession of hazardous operations at hazardous intervals gotten rid of; but it remained for a Bigelow in recent times to devise suitable apparatus, and show the feasibility and safety and great advantage of crushing the stone and withdrawing all its fragments through the natural passage at a single not long sitting. Prof. Fillebrown has devised instruments and accomplished the removal of a piece of nerve trunk in a way so different from the way in which that operation had previously been performed, and possessing such great advantages over it that it is justly to be called a new operation, and we should give him honor.

The cutting out of a piece of nerve trunk for the lasting prevention of the performance of its functions was years ago in a great majority of cases found necessary for success, simple cutting not being usually sufficient. In this connection the injury which the inferior dental nerve may sustain in some rare instances of difficult extraction of lower molars is interesting, and some experience of that kind which I have had I hope to publish sometime. From that experience I should expect after such an operation as Dr. Fillebrown has performed, if relief from neuralgia at once followed, that that relief would be permanent. So far as I have knowledge of these operations, the mobility of the jaw is never in any way interfered with by them, the paralysis induced being purely sensory. I should not expect this incapacity for sensation to remain complete permanently, but I should think that there would always be a lack of full normal sensation,—a difference from that of the other side. I do not believe any collateral nerve supply can ever come to perform the full functions of the nerve from which a cross section of considerable length has been removed. This might be an objection to the operation if it were to be performed for ills of slight and passing moment; but in those cases in which the operation is markedly indicated the relief is so great and comes as such a boon of peace and blessing that the sensory paralysis, limited in extent and partial in degree, is to the patient comparatively a thing of no consequence.

I do not think the diagnosing of cases calling for this operation quite so difficult or uncertain as some of the speakers have intimated. There must be, of course, a most careful exclusion of all such neuroses existing within the teeth themselves as could give rise to the symptoms and be readily and only appropriately treated by less heroic measures—by the ordinary procedures of the dentist. We should endeavor also to exclude those cases of neuralgia which, dependent primarily not upon local lesion, but upon systemic impoverishment, need treatment along a quite different line. With these groups excluded there remains a class of cases most likely explainable by the existence of some such neuroses as may be hopefully traced by the operation of resection, if that resection can be made to reach either the exact seat of the lesion, or to cut between it and the centric portion of the sensory system; and these are cases to which very little hope can be afforded except through resection.

In these cases there are usually such characteristic indications as tend to add positiveness to the diagnosis perhaps first made negatively. In the case of an old gentleman whom I have been seeing in recent months, the teeth from repeated searching examination, including section of some which have been extracted are believed to be intaresically free from any source of offence. After an interval of quiet, paroxysms of agonizing pain are excited by slight movement—the attempt to smile, to speak, to cough, to drink, to take food, the light touch of a finger against the skin of the face, the beard, the moustache, or any other similar part.

While in all of these cases it is desirable to make the diagnosis as accurate and positive as possible, there is a point in nearly all difficult diagnosis which stopping short of completeness extends sufficiently for practical purposes. If we are able to feel pretty confident that the trouble is of a nature to be relieved by resection, the condition of the patient and all other circumstances making no contra-indication, we are justified in making that resection, and all the more so since Prof. Fillebrown has shown how it may be done with no external incision and no after disfigurement. The number of these cases is, to be sure, not larger, and yet I apprehend that few busy dentists pass a single year without seeing one or more instances in which the propriety of this operation is at least a matter for consideration. When they come, if relief may be afforded by it, we should be thankful. Personally I am most grateful to the reader of the paper for the suggestion and the testimony which he has given before us.

*Dr. Williams*—I have read in medical journals descriptions of operations with such instruments as Dr. Fillebrown has mentioned, but they were larger. Dr. Fillebrown was bright enough to have an instrument no larger than he wanted to use.

Dr. Goodwillie, of New York, showed me, at one time, a number of instruments made for him, among them a small trephine and various burrs and cutting instruments. I do not mean to detract at all from Dr. Fillebrown's paper, his ingenuity and success, but I think that we had better not be too forward in claiming things to be new.

*Dr. Fillebrown*—I thank the gentlemen for their kind words of appreciation. I hope it is not entirely unfounded or unmerited. I wish to hedge certainly against having anything claimed for me that I do not claim for myself. All I claim for this is that it is a new method of incising the inferior dental nerve, and that I shall claim right here. I took a great deal of pains; and, among other things, I had this library here gone through, not only in its dental department, but in its surgical department, and I for years have been looking after it, and I haven't been able to find any record where it has been done in anything like the same way.

In the first case the three molars were gone, and I had a grand opportunity to work. In the second case only the second and third molars were gone; the first molar was present, and I performed the operation without injuring that, and through the site of the other teeth.

If I should meet a case where only the third molar was gone, or there were none gone, I should simply remove the third molar, and I think I would cut out three-quarters of an inch.

I should try it, but I should not take the second molar until I had failed in the other case.

*Dr. Taft*—How do you control the bleeding?

*Dr. Fillebrown*—It controls itself. There is another question arises from it; should it persist in bleeding, you would plug the canal of course. But in these cases it didn't; the contraction of the muscles stopped it.

*Dr. Banfield* then presented a regulating appliance which, after placing, had without alteration or change moved a second superior bicuspid into line in twenty-one days. He recommended it for its simplicity and efficiency. *Adjourned.*

